# Prevalence of mutations linked to antimalarial resistance in *Plasmodium falciparum* from Chhattisgarh, Central India: A malaria elimination point of view

**DOI:** 10.1038/s41598-017-16866-5

**Published:** 2017-11-30

**Authors:** Priyanka Patel, Praveen K. Bharti, Devendra Bansal, Nazia A. Ali, Rajive K. Raman, Pradyumna K. Mohapatra, Rakesh Sehgal, Jagadish Mahanta, Ali A. Sultan, Neeru Singh

**Affiliations:** 1 National Institute for Research in Tribal Health, Indian Council of Medical Research, Nagpur Road, Garha, Jabalpur, 482003 Madhya Pradesh India; 2 Department of Microbiology and Immunology, Weill Cornell Medicine - Qatar, Cornell University, Qatar Foundation - Education City, Doha, Qatar; 3 Medical Officer, Community Health Centre Janakpur, District Baikunthpur, Chhattisgarh, India; 4 Regional Medical Research Centre, NE, Indian Council of Medical Research, Post Box no. 105, Dibrugarh, 786 001 Assam India; 5 Department of Medical Parasitology, Postgraduate Institute of Medical Education and Research, Chandigarh, 160012 Punjab India; 6 Present Address: Symbiosis School of Biomedical Sciences, Symbiosis International University, Lavale, Maharashtra 412115 India

## Abstract

Antimalarial drug resistance is a major global challenge in malaria control and elimination. Mutations in six different genes of *Plasmodium falciparum* (*crt*, *mdr1*, *dhfr*, *dhps, ATPase6* and *K-13* propeller) that confer resistance to chloroquine, sulphadoxine-pyrimethamine and artemisinin-based combination therapy were analyzed in samples from Chhattisgarh. Seventy-eight percent of the samples were found to have a *pfcrt* mutation (53% double, 24% triple and 1% single mutant), and 59% of *pfmdr1* genes were found to have an N86Y mutation. Double mutations were recorded in *pfdhfr* gene among 76% of the samples while only 6% of the samples harbored mutant genotypes in *pfdhps*. No mutation was found in the *K-13 propeller* gene, while only one sample showed a mutant genotype for the *PfATPase6* gene. The Tajima test confirmed that there is no role of evolutionary natural selection in drug resistance, and gene pairwise linkage of disequilibrium showed significant intragenic association. The high level of *pfcrt* mutations suggests that parasite resistance to chloroquine is almost at a fixed level, whereas resistance to SP is evolving in the population and parasites remain sensitive to artemisinin derivatives. These findings provide potential information and understanding of the evolution and spread of different drug resistance alleles in Chhattisgarh.

## Introduction

Malaria is a global public health problem, and India alone contributes to 89% of malaria cases in the southeast region^1^. Chhattisgarh State (Central India) is the second most highly malarious state, contributing 14% of the total country’s malaria cases with more than 80% of *Plasmodium falciparum* infections^2^. Over the past decade, the total number of malaria cases and deaths due to malaria has progressively decreased in most of the countries where malaria is endemic^1^. This significant gain has become possible with multisector efforts including the use of artemisinin-based combination therapies (ACT). In India, the combination of artesunate and sulphadoxine–pyrimethamine (AS + SP) is used as a first-line treatment for uncomplicated *P. falciparum* malaria except in the northeastern states where artemether + lumefantrine is used. Furthermore, a recent rise in ACT resistance against *P. falciparum* in Cambodia and neighboring countries poses a serious threat to malaria control and elimination globally and in India^3,4^. Considering the evolution and spread of malaria, parasites resistant to different antimalarials such as chloroquine (CQ), sulphadoxine-pyrimethamine (SP), and an artemisinin derivative (ART) influence malaria’s epidemiological outcome and present a great challenge to malaria control programs^5–7^. Resistance to chloroquine has been found to be associated with mutations at several amino acid positions in the *P. falciparum* chloroquine-resistance transporter (*pfcrt*) and *P. falciparum* multidrug resistance (*pfmdr1*) genes^8^. Point mutations at codons 72–76 of *pfcrt* and N86Y, Y184F in *pfmdr1* are known for chloroquine resistance^9,10^. Antifolate resistance has been associated with point mutations in the dihydrofolate reductase (*pfdhfr*) and dihydropteroate synthase (*pfdhps*) genes^11–13^. Point mutations at codons 16, 51, 59, 108 and 164 of *pfdhfr* inhibit its activity, and the parasite becomes resistant to pyrimethamine, while mutations at 436, 437, 540, 580 and 613 of *pfdhps* reduce the substrate binding capacity and confer resistance to sulphadoxine^14^.

Polymorphisms of the sarcoplasmic/endoplasmic reticulum Ca2+-ATPase ortholog in *P. falciparum* (*PfATPase6*) have been associated with ART resistance although the association of SNPs in *pfatpase6* with resistance to ART is not yet fully confirmed^15,16^. However, recently, *K-13 propeller* (Pf3D7-1343700 kelch propeller) as a key molecular marker for ART resistance has been reported^17^. Tracking the pattern of mutations, estimating the single nucleotide polymorphism (SNP) level and assessing linkage among the SNPs in the population are the most efficient ways to understand the evolution of a particular gene^18^. Therefore, to achieve the goal of malaria elimination, molecular data on anti-malarial drug resistance with wide coverage in India, particularly in highly endemic states, is needed for proper implementation of antimalarial drug treatment policy. With this aim, we assessed the prevalence of point mutations involved in the antimalarial resistance genes *pfcrt* & *pfmdr1* (for CQ), *pfdhfr* & *pfdhps* (for SP) and *PfATPase6* & *K-13 propeller* (for ART) among *P. falciparum* samples from Chhattisgarh, Central India. The molecular data from this study could contribute to the comprehensive baseline information on antimalarial drug resistance prior to a malaria elimination phase in India.

## Results

### Demographic profile of the study population

A total of 6718 patients were screened; of these patients, 5.2% (n = 352) were found to be positive for malaria parasite {*P. falciparum* (n = 271), *P. vivax* (n = 79) or a mixed *P. falciparum* and *P. vivax* infection (n = 2)}. Of 271 *P*. *falciparum* patients, 180 polymerase chain reaction (PCR)-positive patients who fulfilled the enrolment criteria were included in the study (Table 1). The majority of the participants were from the tribal community (75%, n = 134).

**Table 1:** Demographic characteristics of the study patients.

Parameter	Value (n = 180)
Sex [no (%)]	
Male	87 (48.33)
Female	93 (51.67)
Age Category [no(%)] [mean ± SD]	
0–4 Years	24 (13.33)
	2.92 ± 0.19
>4–8 years	24 (13.33)
	6.63 ± 0.22
>8–14 Years	31 (17.22)
	11.55 ± 0.23
>14 Years	101 (56.11) 30.44 ± 1.29
Parasite Count [no/µl]	
Mean	5827.05
Range	31.9–176720
SD	±17294.85
95% CI	3283.29–8370.80

### Molecular analysis of *pfcrt* & *pfmdr1* genes

**Figure 1:**
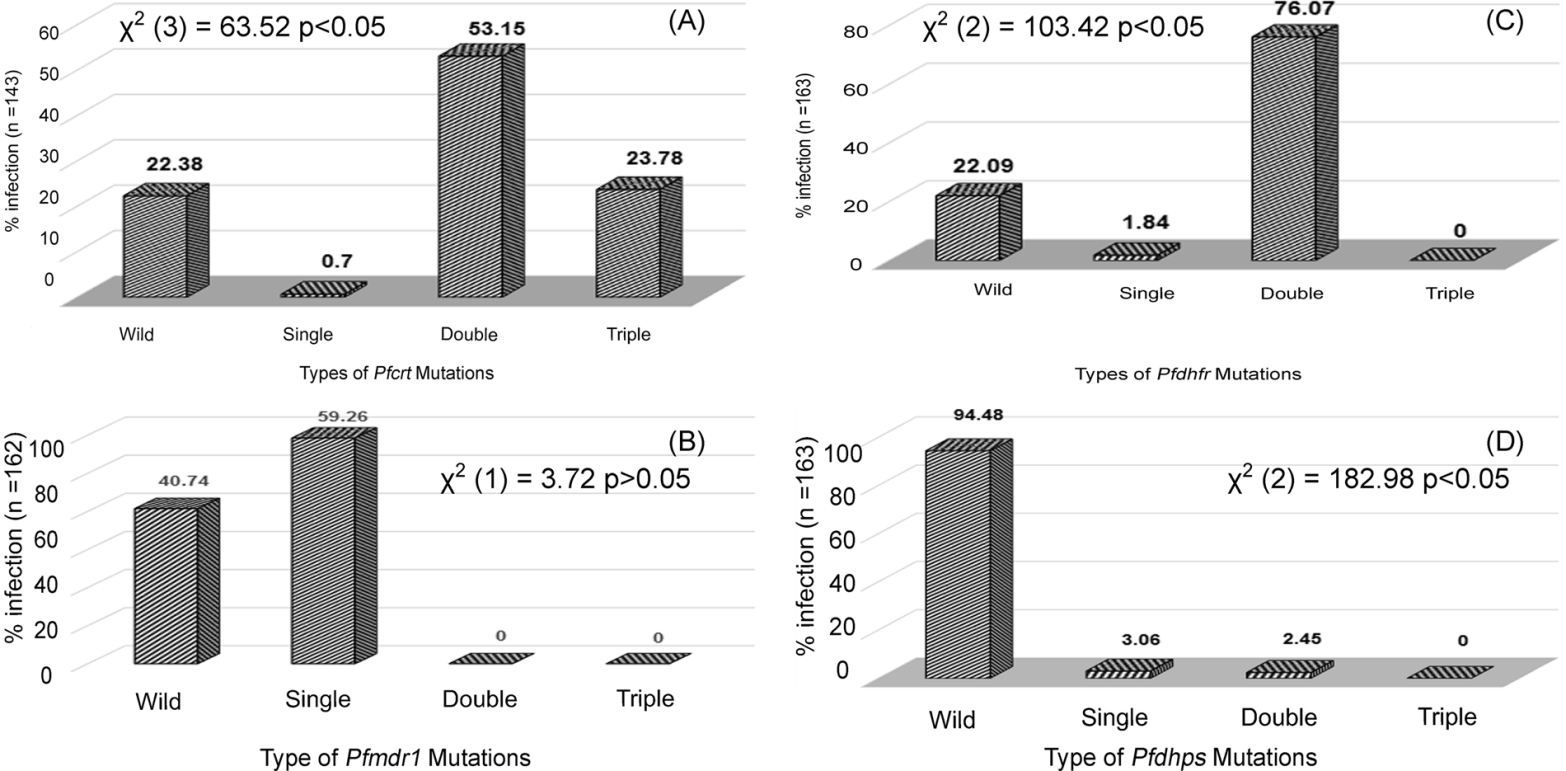
Mutation rates in *P. falciparum* genes (*Pfcrt, Pfdhfr, Pfdhps* and *Pfmdr1*) that confer resistance to antimalarials.

DNA was isolated from all the enrolled samples (n = 180), and analysis of *pfcrt*, *pfmdr1, pfdhfr, pfdhps, PfATPase6* and *K-13* propeller mutations was attempted using DNA sequencing methodology. A total of 143 samples were successfully analyzed for the *pfcrt* gene covering codons 44–177. Out of these, 78% of the samples were mutant genotypes, while only 22% were wild type (Table 2). In addition, codon K76T was found as a mutant position in all the mutant genotypes (NCBI GenBank database accession KY862117- KY862119). Fifty-three percent of isolates were found to be a double mutant at positions C72S and K76T followed by 24% triple mutants at codons M74I, N75E, K76T, and a single point mutation at K76T was observed in only 1% of samples (Fig. 1).

**Table 2:** Mutation analysis of *pfcrt, pfmdr1, pfdhfr, pfdhps*, *pfATPase6*, and *K13* genes in the study population.

Candidate gene	Occurrence of mutation		Haplotype diversity	Nucleotide diversity		Test of neutrality Tajima’s D
	N (%)	95% CI		π	Ɵ	
*pfcrt* (n = 143)			0.615	0.01102	0.00987	0.28315
C_72_V_73_M_74_N_75_K_76_	32 (22.38)	15.84–30.10				
C_72_V_73_M_74_N_75_ **T**_76_	1 (0.70)	0.02–3.84				
**S**_72_V_73_M_74_N_75_ **T**_76_	76 (53.15)	44.63–61.53				
C_72_V_73_ **I**_74_ **E**_75_ **T**_76_	34 (23.78)	17.06–31.60				
*pfmdr1* (n = 162)			0.486	0.00108	0.00039	1.83461
N_86_	66 (40.74)	33.10–48.73				
**Y**_86_	96 (59.26)	51.27–66.90				
*pfdhfr* (n = 163)			0.375	0.00122	0.00060	1.43780
A_16_N_51_C_59_S_108_I_164_	36 (22.09)	15.97–29.24				
A_16_N_51_ **R**_59_S_108_I_164_	1 (0.61)	0.02–3.37				
A_16_N_51_C_59_ **N**_108_I_164_	2 (1.27)	0.15–4.36				
A_16_N_51_ **R**_59_ **N**_108_I_164_	124 (76.07)	68.78–82.40				
*pfdhps* (n = 163)			0.106	0.00038	0.00100	−1.02426
S_436_A_437_K_540_A_581_A_613_	154 (94.48)	89.78–97.45				
S_436_ **G**_437_K_540_A_581_A_613_	5 (3.06)	1.00–7.01				
**A**_436_G_437_ **E**_540_A_581_A_613_	4 (2.45)	0.67–6.16				
*PfATPase6* (n = 143)			0.014	0.00004	0.00048	−0.98824
Wild-type	142 (99.30)	96.17–99.98				
Non-synonymous mutation						
A2167G (I723V)	1 (0.70)	0.02–3.84				
*K13* (n = 143)			—	—	—	—
Wild-type	143 (100.0)	—

**Figure 2:**
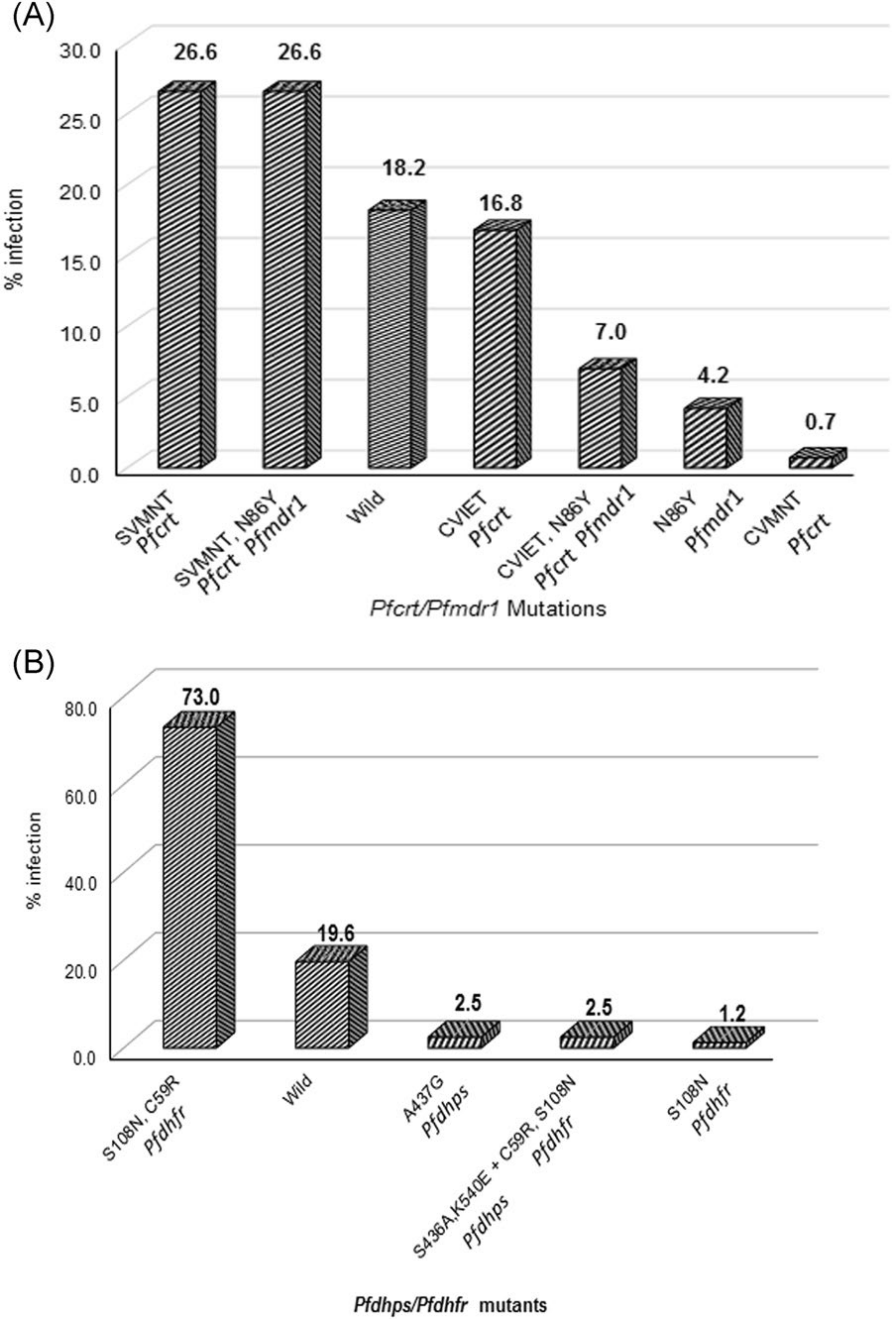
(**A**) Prevalence of grouped *Pfcrt/Pfmdr1* alleles in *P. falciparum* linked to chloroquine resistance from central India. (**B**) Prevalence of grouped *Pfdhps/Pfdhfr* alleles in *P. falciparum* linked to sulphadoxine-pyrimethamine resistance from central India.

The *pfmdr1* gene was amplified and analyzed from 162 samples, and 59% were found mutant at N86Y (Table 2). Combining the *pfcrt* and *pfmdr1* mutations, only 18% of samples were found to be wild-type, and the majority (82%) were mutant genotypes (Fig. 2). Additionally, 27% of the samples harbored a double mutant (SVMNT) *pfcrt* genotype with mutant *pfmdr1*, and 7% had a triple mutant (CVIET) genotype with mutant *pfmdr1* (NCBI GenBank database accession KY862127- KY862128).

### Analysis of *pfdhfr* and *pfdhps* mutations

A total of 163 *P*. *falciparum* samples were successfully amplified and analyzed for both the *pfdhfr* and *pfdhps* genes. Out of the five *pfdhfr* codon mutations (A16V, N51I, C59R, S108N/T and I164l) conferring pyrimethamine resistance, only two, C59R and S108N, were found in this study either as single mutants or in the form of a double mutant (Table 2). Only 22% of samples were found to be the wild-type genotype (A_16_N_51_C_59_S_108_I_164_). The majority (76%, n = 124) of samples were double mutants and only 2% (n = 3) of samples were single mutants (Fig. 1). A total 4 haplotypes were determined with haplotype diversity of 0.375 (NCBI GenBank database accession KY862120- KY862122). Out of the five *pfdhps* mutations (S436F/A, A437G, K540E, A581G AND A613 S/T) known to be involved in sulphadoxine resistance, three codons, S436A, A437G and K540E, were found to be mutant either as single or double mutants (Table 2). A low level (6%) of mutant genotypes (3% single and 3% double mutant) (Fig. 1) and a total of 3 haplotypes were found with 0.106 haplotype diversity (NCBI GenBank database accession KY862123- KY862126). Furthermore, the combined results for both *pfdhfr* and *pfdhps* showed that only 20% of samples were wild type. Quadruple mutations of *pfdhfr* (59,108) and *pfdhps* (436,540) were found in only 3% of isolates (Fig. 2).

### Analysis of *PfATPase6* and K-13 mutations

The *PfATPase6* gene was successfully sequenced from the PCR products of 143 samples (79%, n = 143/180) and was analyzed by comparing with the reference strain 3D7 (Pf3D7_0106300) covering codons 653–797. The known SNPs, N683K and S769N substitutions, associated with ART resistance in the *pfATPase6* gene were absent and genotyped as wild type (NCBI GenBank database accession KY862115-KY862116). However, only one sample with a non-synonymous mutation was found at nucleotide position A2167G, which covers codon I723V (Table 2). Additionally, the propeller region of the *K-13* gene was successfully sequenced from 143*P. falciparum* samples. No polymorphism was found in any of the 17 locations in the *K-13 propeller* region covering codons 427–702 that confer resistance to ART (NCBI GenBank database accession KY862114).

**Figure 3:**
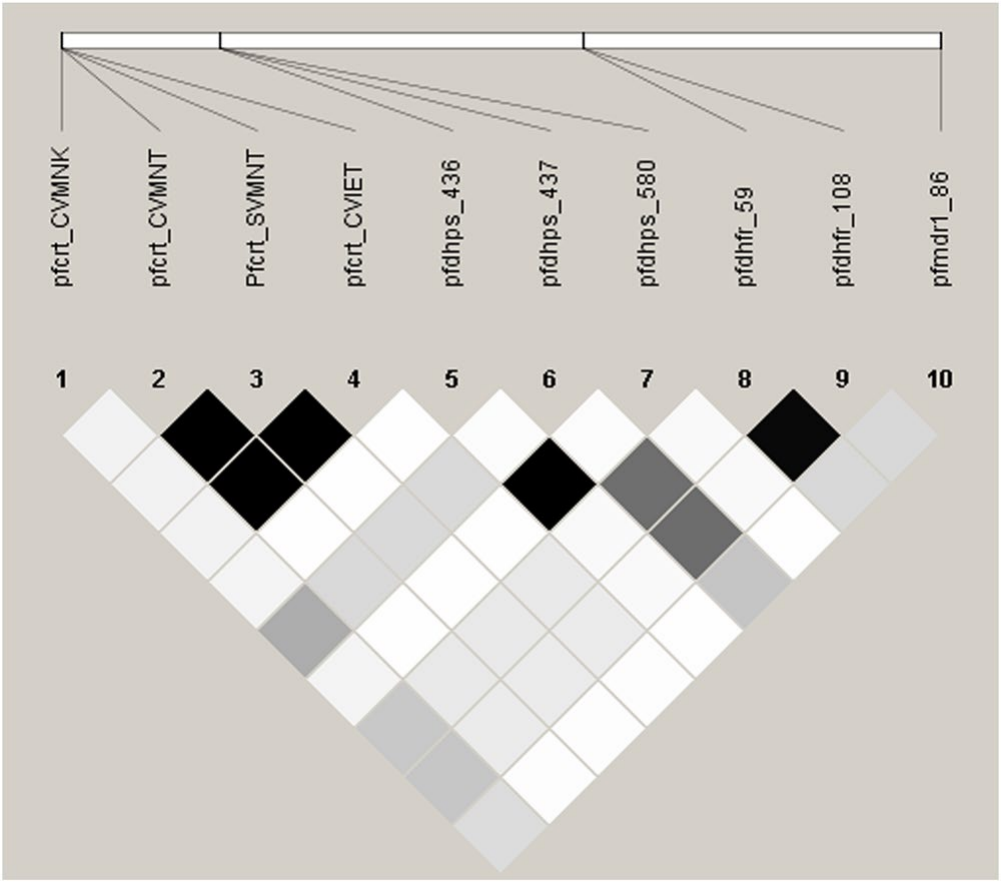
Linkage disequilibrium (LD) between pairs of SNPs located in four different genes (*Pfcrt, Pfdhfr, Pfdhps* and *Pfmdr1*) implicated in drug resistance in *P. falciparum* populations of Central India. The strength of LD between the SNPs was determined from the association of statistical significance by calculating the r^2^ values and represented by the darkness of the boxes.

Furthermore, we extended our analysis, and Tajima’s D test of neutrality confirmed that there was no role of evolutionary natural selection in the drug resistance genes. However, pairwise LD estimation to assess the selection of *P. falciparum* resistance markers revealed a total of 34 possible pairs of SNPs with fine statistically significant intragenic associations, but no intragenic associations were found between different genes (Fig. 3).

## Discussion

Drug resistance in *P. falciparum* malaria has become an important hurdle for malaria control and elimination globally. In 2010, ACT was implemented as a first-line treatment for uncomplicated malaria in India. Although artemisinin (ART) resistance has not appeared in India, its presence in Myanmar, 25 km away from the Indian border^4^, generates concern about drug-resistant strains following the same historical corridor and spreading into India, as seen previously with other anti-malarials^19^. Hence, continuous monitoring or molecular surveillance of drug resistance is very important to provide early warning and to guide national drug policies. The emergence of drug-resistant parasites in India is quite alarming, as drug resistance against CQ and SP has already been circulating in the northeast and other parts of the country^5,6,19–21^. The availability of easy and rapid molecular methods is extremely useful for the detection of drug resistance and plays an important role in epidemiological surveys as well as in regular updating of antimalarial drug policy. We investigated mutations in the *pfcrt*, *pfmdr1, pfdhfr, pfdhps, PfATPase6* and *K-13 propeller* genes of *P. falciparum* samples collected from Chhattisgarh, Central India, to determine the current extent of resistance to CQ, SP, and ART. A number of recent studies have shown reoccurrence of CQ sensitivity among *P. falciparum* populations after its withdrawal^22–24^. In the present study, we found four different haplotypes including 22% wild type (CQ sensitive), which is rarely observed throughout the country^25–27^. However, our findings corroborate recent reports (after 6 years of CQ withdrawal) where 20–40% of samples carried a wild-type haplotype^28,29^, suggesting that CQ-sensitive *P. falciparum* strains might have returned after the withdrawal of CQ. In this study only one case of single point mutation (C_72_V_73_M_74_N_75_ **T**_**76**_) were found while majority of the cases (53%) were double mutant genotype (**S**_**72**_V_73_M_74_N_75_ **T**_**76**_) which is already established to be the chloroquine resistant genotype^5^. We also found that 24% of samples carried triple mutations (C_72_V_73_ **I**_74_ **E**_75_ **T**_76_ haplotype) known to confer higher resistance to CQ, suggesting a high level of CQ resistance still in the *P. falciparum* parasite population in this region. Moreover, in the *pfmdr1* gene, a mutation at codon N86Y is known to contribute to CQ resistance^30^, but the *pfmdr1* gene has been found to correlate poorly with CQ resistance^31^. The prevalence of the N86Y mutation varies from 0 to 100% across the country^21,27,29,32,33^. In this study, 35% of samples had a mutant genotype of the *pfcrt* gene along with an N86Y *pfmdr1* mutant genotype, which agrees with Djimde *et al*. 2001 who also reported that the N86Y mutation modulates a higher level of CQ resistance when present with the mutant K76T *pfcrt* genotype^34^. Mutations in the *pfdhfr* and *pfdhps* genes are associated with SP resistance and have been reported in most parts of the country^25,26,35–38^. In this study, a high level (78%) of the *pfdhfr* mutation (A_16_N_51_ **R**_59_S_108_I_164,_ A_16_N_51_C_59_ **N**_108_I_164,_ A_16_N_51_ **R**_59_ **N**_108_I_164_) and a low level (6%) of *pfdhps* mutations (S_436_ **G**_437_K_540_A_581_A_613,_ **A**_436_G_437_ **E**_540_A_581_A_613_) was recorded. This suggests a moderate degree of reduced susceptibility to SP and a low risk of treatment failure at the time of the study, as the authors have assessed the efficacy of artesunate + sulphadoxine–pyrimethamine against uncomplicated *P. falciparum* and found 100% efficacy (unpublished data). Single point mutation among these genes is the only early sign of the improper action of the drugs, while double mutation (A_16_N_51_ **R**_**59**_ **N**_**108**_I_164_) showed that the sensitivity of parasite against drug is decreasing and it might be responsible for drug resistance. However, the triple (A_16_N_51_ **R**_**59**_ **N**_**108**_ **L**_**164**_) and quadruple (A_16_ **A**_**51**_ **R**_**59**_ **N**_**108**_I_164_) mutation in the *Pfdhfr* gene conferred the resistance against the drugs. In the presesent study, 3% of samples had a quadruple mutation (*pfdhfr* 59 + 108 and *pfdhps* 436 + 540), indicating that these parasite populations need one more SNP in *pfdhfr* to become resistant *P. falciparum* haplotypes^39^. It is worth mentioning that we did not find a single case of triple mutation in either the *pfdhfr* or *pfdhps* gene despite the high rate of malaria transmission in the region. ART resistance is considered a major risk to public health with the hazard of ART resistant parasites spreading from western Cambodia to the greater Mekong sub-region^17^. However, in India, artemisinin derivatives remain quite effective in treating malaria, but the presence of non-synonymous mutations in the propeller region and decreased drug efficacy are important deterrents in the fight against malaria^6,40–43^. Mutations in *K-13 propeller* have been identified as an important and putative molecular marker for ART resistance. Currently, 108 non-synonymous mutations from different geographic regions of the world^44^ and four point mutations, i.e., Y493H, R539T, I543T and C580Y, have exhibited associations with ART resistance^17^. In addition, 13 other point mutations have also been associated with late parasite clearance^3,45,46^. Recently, *K-13 propeller* mutations have been reported in southeast countries^44^ and Bangladesh^47^. However, in India, very limited polymorphism has been reported^7,42^; in the present study, all samples were found to be wild type, which agrees with a report from the West Bengal region^48^. Interestingly, in the *PfATPase6* gene, one sample had a non-synonymous mutation at the I723V position, which is consistent with previous reports from India and Equatorial Guinea^39,49^.

Furthermore, in the present study, the π value of nucleotide diversity was higher in the *pfcrt* gene and lower than that in the *PfATPase6* gene. This confirmed that mutant alleles are fixed in the population; Chauhan *et al*. 2014 and Antony *et al*. 2016 reported similar findings among Indian isolates^29,33^. These data confirm that resistance against SP is low while resistance against ART was not observed in this study. We observed significant intragenic association among the *pfcrt, pfdhfr* and *pfdhps* genes, which is in agreement with previous reports from Odisha and Pondicherry states^18,29^. Additionally, no significant intergenic association was found in the SNPs of different genes; this finding, however, contrasts with Kar *et al*. 2016 who found strong intergenic associations with the *pfcrt* gene in combination with the *pfmdr*, *pfdhfr* and *pfdhps* genes^28^. Therefore, further large-scale genetic population studies are required to compare the LD association of different endemic malaria regions. The genotyping of drug resistance markers can reveal the efficacy of current treatment regime. Therefore, the data generated from this study can be used to improve the patient care and disease management and help researchers working on clinical efficacy of different antimalarials.

The principle limitations of this study are that (i) we did not carry out an *in vivo* therapeutic efficacy study from the same site and that (ii) we did not correlate the clinical outcome with mutation patterns. However, there is no obvious bias in the sample collection, and these comprehensive molecular data are a representation of both past and present antimalarial drugs. The strengths of this study is the comprehensive use of sensitive and standardized assays, rendering our findings amenable to a detailed analysis of genetic polymorphisms responsible for antimalarial *P*. *falciparum* resistance genes from an area where malaria is a major health problem. In the present study, we aimed for such analysis to be relevant for epidemiologic investigations, and design of control measures for malaria control and subsequent to elimination.

## Methods

### Study area, population and sample collection

This study was carried out at Janakpur Community Health Care (CHC), district Baikunthpur, Chhattisgarh, Central India (23.7191°N, 81.7883°E and 550 M height above sea level). This is a secondary health care facility situated in a remote area of the district surrounded by dense forest (60%), and the majority (65%) of the population work mainly in forest nurseries and use traditional folk medicine for their treatment. *P. falciparum* is the predominant species followed by *P. vivax*, and both *Anopheles culicifacies* and *A. fluviatilis* are responsible for transmitting the disease in this area. The present study was carried out from August 2013 to March 2015, and symptomatic patients were screened for malaria parasites by microscopy using thick and thin blood smears stained with JSB stain. Patients having asexual stage of *P. falciparum* monoinfection with no other symptoms of severe malaria were included in this study. Patients have chronic infection of any other disease were excluded from the study. Patients were given the treatment with Artemisinin combination therapy (Artesunate plus sulfadoxine-pyrimethamine) as per national guideline (National Vector Borne Disease Control Programme) and this combination therapy is 100% efficacious for uncomplicated *P. falciparum* malaria in this region. Intravenous blood samples, in sterile conditions, were collected from the patients positive for *P. falciparum* malaria after taking their written informed consent.

### Ethical approval

The study protocol for patient participation and collection of blood samples for laboratory testing was reviewed and approved by the institutional ethics committee of NIRTH, Jabalpur. All methods were performed in accordance with the relevant guidelines and regulations. All study participants provided written informed consent prior to their participation according to Indian Council of Medical Research (ICMR), New Delhi, India guidelines. A copy of the consent form in the local language was also provided and explained to the patients or the parents/guardian of children. The participation of other institutes was also approved by the ICMR.

### Genomic DNA extraction and parasite genotyping

Genomic DNA was isolated by QIAamp DNA blood mini kit as per the manufacturer’s instructions (Qiagen, CA, USA) and stored at −20 °C for further use.

The K76T and N86Y mutations in the *pfcrt* and *pfmdr1* genes, respectively, which are the primary determinant markers of CQ resistance, were targeted using nested PCR and Sanger sequencing. The *pfcrt* region covering codons 44–177 (582 bp) was performed as described earlier^5^. The *pfmdr1* region covering codons 1–177 (521 bp) was sequenced as per protocol described earlier by Djimde *et al*. 2001^34^. Alleles of the *pfdhfr* and *pfdhps* genes, primary molecular markers for sulphadoxine-pyrimethamine resistance, were amplified and analyzed as described previously^35,50^. The PCR amplification of the *pfdhfr* gene spanning codons 51–164 and *pfdhps* gene spanning codons 425–640 was carried out and the details of nested PCR primers and cycling conditions are given in the supplementary table. In brief, primary PCR was performed in a volume of 25 μL with 0.2 U of *Taq* polymerase enzyme (Invitrogen, life technologies), 0.2 mM each dNTP, 1 μM each primer and 1.5 mM MgCl_2._ The newly discovered molecular markers *K-13 propeller* ^17^ and *PfATPase6* for tracking the emergence and spread of ART resistance in *P. falciparum* were determined. The *PfATPase6* gene was amplified using single-step PCR covering codons 653–797 as described by Zhang G *et al*. 2008^51^. In this study all the amplicon sizes were less than 1Kb and positive control samples always provided correct sequences.

### Nucleotide sequencing

The PCR products were purified from the agarose gel using a Hiyield^TM^ gel/PCR DNA extraction kit according to the manufacturer’s recommended protocol. The gel-purified product was used with the ABI Big dye Terminator Ready Reaction Kit Version 3.1 for sequencing PCR. The sequencing PCR was performed in a volume of 20 μl with 1 μl of Terminator Ready Reaction Mix (TRR), 3.2 pmol of gene specific primer and 5X sequencing buffer. The cycling conditions for the sequencing PCR include 25 cycles of denaturation at 96 °C for 10 sec, annealing at 50 °C for 5 sec and extension at 60 °C for 4 min. The sequences were aligned and analyzed using Bio edit Sequence Alignment Editor v.7.0.5.2 software and online sequence alignment tool Clustal W.

### Statistical analysis

Data were entered in Microsoft Excel and exported to Stata version 12.0 for analysis. Chi-square and Fischer exact tests were used where applicable to assess the relationship between single and multiple mutations. In addition, haplotype diversity^52^ and nucleotide diversity (Ɵ & π) were estimated for each gene. The nucleotide diversity parameter π is estimated based on the average number of pairwise nucleotide differences per site^53^ and Ɵ estimates are dependent on the number of segregating sites. All the parameters were estimated using DnaSP version 5.10.01. To determine the association between SNPs (present in *pfcrt, pfmdr1, pfdhfr* and *pfdhps)* in the population, both inter- and intragenic linkage disequilibrium (LD) tests were performed using the Haploview Software^54^.

## Conclusion

This was the first comprehensive molecular study carried out in this geographical area focusing on mutations of the *pfcrt, pfmdr1*, *pfdhfr*, *pfdhps*, *PfATPase6* and *K-13 propeller* genes that were strongly associated with CQ, SP and ART resistance. This study showed a high level of CQ resistance genotypes, a moderate level of SP resistance and evolving SP resistance genotypes. Fortunately, no mutations were found against ART resistance.

### Data availability

All data generated or analyzed during this study are included in this published article.

## Electronic supplementary material

Table S1
