# Supplementary material for: Prevalence of mutations linked to antimalarial resistance in Plasmodium falciparum from Chhattisgarh, Central India: A malaria elimination point of view
Source: Sci Rep. 2017 Nov 30;7:16690. doi: 10.1038/s41598-017-16866-5 (PMC5709362; doi:10.1038/s41598-017-16866-5)
Supplement: Supplementary file 1 — Table S1 [file 41598_2017_16866_MOESM1_ESM.pdf]

Prevalence of mutations linked to antimalarial resistance in *Plasmodium falciparum* from Chhattisgarh, Central India: A Malaria elimination point of view

Priyanka Patel[1#], Praveen K. Bharti[1], Devendra Bansal[2], Nazia A Ali[1], Rajive K. Raman[3], Pradyumna K. Mohapatra[4], Rakesh Sehgal[5], Jagadish Mahanta[4], Ali A. Sultan[2], Neeru Singh[1]

[1]National Institute for Research in Tribal Health, Indian Council of Medical Research, Nagpur Road, Garha, Jabalpur - 482003, Madhya Pradesh, India

[#]Symbiosis School of Biomedical Sciences, Symbiosis International University, Lavale, Maharashtra 412115, India

[2]Department of Microbiology and Immunology, Weill Cornell Medicine - Qatar, Cornell University, Qatar Foundation - Education City, Doha, Qatar.

[3]Medical Officer, Community Health Centre Janakpur, District Baikunthpur, Chhattisgarh, India

[4]Regional Medical Research Centre, NE, Indian Council of Medical Research, Post Box no. 105, Dibrugarh - 786 001, Assam, India

[5]Department of Medical Parasitology, Postgraduate Institute of Medical Education and Research, Chandigarh - 160012, Punjab, India

[*]Corresponding Author

Dr. Neeru Singh

Director, National Institute for Research in Tribal Health

(ICMR) Nagpur Road Garha, Jabalpur-482003, Madhya

Pradesh, India

Email – neeru.singh@gmail.com

Supplementary Information

Table S1: Primer Sequence and PCR condition used for amplification of *P. falciparum* Drug resistance genes

Gene	Primer name	Primer Sequence	PCR product Length (bp)	Denaturation	Annealing	Elongation	No of Cycles	Ref
*pfcrt*_Primary	PFCF	CCGTTAATAATAAATACAGGCAG	1.6 kbp	95 °C, 30 sec	54 °C, 1 min	60 °C, 1 min	40	Bharti et al 2010(5)
	PFCR	CTTTTAAAAATGGAAGGGTGTATAC	1.6 kbp	95 °C, 30 sec	54 °C, 1 min	60°C, 1 min		
*pfcrt*_Nested	PF72	TGTGCTCATGTGTTTAAACTTAT	582 bp	95 °C, 30 sec	50 °C, 1 min	60 °C, 1 min	30	
	PR72	AAAATAGTATACTTACCTATATCT	582 bp	95 °C, 30 sec	50 °C, 1 min	60°C, 1 min		
pfmdr1 Primary	MDR1	ATGGGTAAAGAGCAGAAAGA	603 bp	95 °C, 1 min	48°C, 1 min	72 °C, 1 min	**35**	Djimde et al 2001(34)
	MDR2	AACGCAAGTAATACATAAAGTCA	603 bp	95 °C, 1 min	48°C, 1 min	72 °C, 1 min		
pfmdr1 Nested	MDR3	TGGTAACCTCAGTATCAAAGAA	521 bp	95 °C, 1 min	48 °C, 1 min	72 °C, 1 min	**30**	
	MDR4	ATAAACCTAAAAAGGAACTGG	521 bp	95 °C, 1 min	48 °C, 1 min	72 °C, 1 min		
pfdhfr Primary	PF1	TTTATATTTTCTCCTTTTTA	718 bp	95 °C, 1 min	45°C, 45 sec	72 °C, 1 min	35	Ahmed A et al 2004, 2006 (35,50)
	PR1	CATTTTATTATTCGTTTTCT	718 bp	95 °C, 1 min	45°C, 45 sec	72 °C, 1 min		
pfdhfr Nested	PF1	TTTATATTTTCTCCTTTTTA	648 bp	95 °C, 1 min	45°C, 45 sec	72 °C, 1 min	30	
	NR2	ACAGAAATAATTTGATACTCA	648 bp	95 °C, 1 min	45°C, 45 sec	72 °C, 1 min		
pfdhps Primary	F1	CCATTCCTCATGTGTATACAACAC	1330 bp	95 °C, 1 min	55 °C, 45 sec	72 °C, 1 min	35	Ahmed A et al 2004 (35)
	R1	CTTGGTCTATTTTTGTTAAAACATCC	1330 bp	95 °C, 1 min	55 °C, 45 sec	72 °C, 1 min		
pfdhps Nested	F2	TGGAATATTAAATGTTAATTATGA	653 bp	94 °C, 1 min	50 °C, 45 sec	72 °C, 1 min	30	
	R2	TTTTCATTTTGTTGTTCATCATGT	653 bp	94 °C, 1 min	50 °C, 45 sec	72 °C, 1 min		
pfatpase6	F2	AAAATAAATACCACATCAACACAT	437 bp	95 °C, 1 min	50 °C, 50 sec	72 °C, 1 min	35	Zhang G (51)
	R2	TCAATAATACCTAATCCACCTAAA	437 bp	95 °C, 1 min	50 °C, 50 sec	72 °C, 1 min		
K 13 Propeller	K13_F	GCCAAGCTGCCATTCATTTG	849bp	95 °C, 45 sec	62 °C, 1 min. 50 sec	72 °C, 1 min	30	Ariey et.al. 2014 (17)
	K13_R	GCCTTGTTGAAAGAAGCAGA	849bp	95 °C, 45 sec	62 °C, 1 min. 50 sec	72 °C, 1 min	30	
